# High-Quality PDF Data Collection with a Ag source Single Crystal X-ray Diffractometer

**DOI:** 10.1063/4.0000903

**Published:** 2025-10-27

**Authors:** Ashley (Weiland) Schmidt

**Affiliations:** Bruker AXS, Madison, WI, USA

## Abstract

Revolutionize your research with cutting-edge Pair Distribution Function (PDF) measurements using a Single Crystal X-ray diffractometer. This presentation offers a comprehensive overview of PDF measurements, emphasizing in- house diffraction techniques using a state-of-the-art Ag radiation source to collect high-quality, high Q data.

Attendees will be guided through experimental setup, highlighting key steps and best practices to ensure accurate data collection. The talk will then delve into the analysis phase, demonstrating how to generate PDF patterns. Example cases will be explored throughout each step.

